# Designing Multi-Dopant Species in Microporous Architectures to Probe Reaction Pathways in Solid-Acid Catalysis

**DOI:** 10.3389/fchem.2020.00171

**Published:** 2020-03-17

**Authors:** Matthew E. Potter, Lindsay-Marie Armstrong, Marina Carravetta, Thomas M. Mezza, Robert Raja

**Affiliations:** ^1^Faculty of Engineering and Physical Sciences, University of Southampton, Southampton, United Kingdom; ^2^UOP, A Honeywell Company, Des Plaines, IL, United States

**Keywords:** catalysis, zeotypes, solid-acid, ethanol, CFD

## Abstract

The introduction of two distinct dopants in a microporous zeotype framework can lead to the formation of isolated, or complementary catalytically active sites. Careful selection of dopants and framework topology can facilitate enhancements in catalysts efficiency in a range of reaction pathways, leading to the use of sustainable precursors (bioethanol) for plastic production. In this work we describe our unique synthetic design procedure for creating a multi-dopant solid-acid catalyst (MgSiAPO-34), designed to improve and contrast with the performance of SiAPO-34 (mono-dopant analog), for the dehydration of ethanol to ethylene. We employ a range of characterization techniques to explore the influence of magnesium substitution, with specific attention to the acidity of the framework. Through a combined catalysis, kinetic analysis and computational fluid dynamics (CFD) study we explore the reaction pathway of the system, with emphasis on the improvements facilitated by the multi-dopant MgSiAPO-34 species. The experimental data supports the validation of the CFD results across a range of operating conditions; both of which supports our hypothesis that the presence of the multi-dopant solid acid centers enhances the catalytic performance. Furthermore, the development of a robust computational model, capable of exploring chemical catalytic flows within a reactor system, affords further avenues for enhancing reactor engineering and process optimisation, toward improved ethylene yields, under mild conditions.

## Introduction

**Figure 1:**
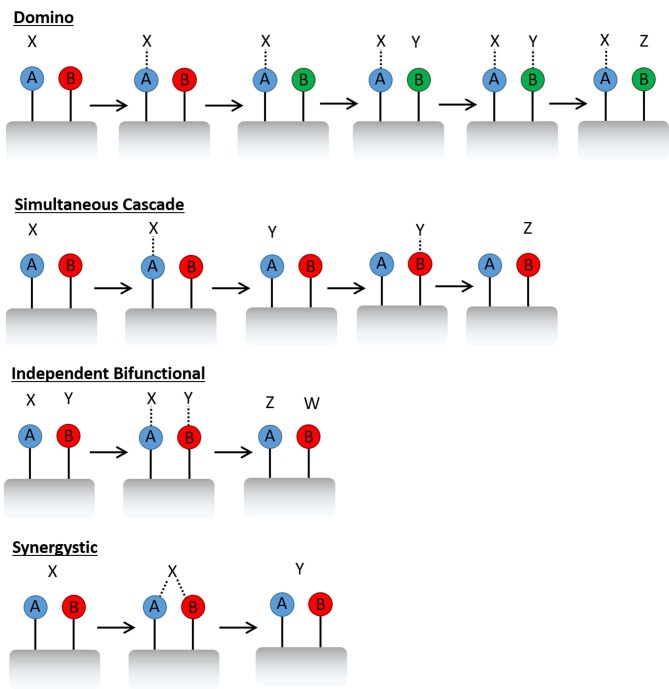
Schematic showing different bimetallic reaction pathways.

Rational catalytic design is an emerging theme that enables the targeted discovery of single-site heterogeneous catalysts (Thomas et al., 2005) that can be tailored for chemical applications, by dextrous manipulation of active sites within framework architectures. Many examples exist, where subtle modifications to a material, such as a change of active-site precursor, or variation in synthesis conditions, have facilitated significant catalytic improvements (Munnik et al., 2015; Rogers et al., 2017; Li Y. et al., 2018). While many systems have benefited from this type of synthetic optimisation, a large proportion of catalysts have been improved by the addition of a second metal (Thomas and Raja, 2005; Huo et al., 2011; Alonso et al., 2012; Villa et al., 2015; Xiao and Varma, 2018). Metallic promoters are common place in industry, often used to improve the catalysts lifetime, making it less susceptible to coking or sintering (De et al., 2016). Though a second metal site also offers a range of catalytic possibilities in multi-step catalysis, such as the creation of bifunctional materials for domino or simultaneous cascade reactions (Figure 1) (Zeidan et al., 2006; Paterson et al., 2011; Bui et al., 2013). In such processes, one active site will form an intermediate, which either triggers the next active site (domino) (Bui et al., 2013) or results in a product which initiates the next process (simultaneous cascade) (Zeidan et al., 2006). The active site can also be designed in such a way that two metals perform complementary roles, where either, each active site performs an unique role in a concerted fashion, or can synergistically enhance the same role (Leithall et al., 2013).

In all cases the precise proximity of the two metals, at the atomic level, is vital for engineering improved catalytic behavior, thus must be carefully controlled (Leithall et al., 2013; Potter et al., 2015). Creating multi-dopant entities is often trivial, and readily achieved through simplistic impregnation and deposition processes; however this seldom gives predictive control over the relative locations of the two metals (Jiang et al., 2015). A range of synthetic techniques can promote interactions between the different metals, this is particularly true in nanoparticle design, where core-shell nanoparticles encourage partial mixing of two different metals (Price et al., 2011). Similarly alloyed multi-dopant nanoparticles can be synthesized (Hermans et al., 2001; Raja et al., 2001; Hungria et al., 2006; Adams et al., 2013) *in situ* or formed through precursors complexes such as Ir_3_(CO)_9_(μ_3_-Bi) which decomposes to yield Ir_3_Bi nanoparticles on a suitable support (Adams et al., 2013). While elegantly designed, such metallic nanoparticles are often prone to oxidation, agglomeration and sintering under intense reaction conditions. In contrast, isomorphous framework substitution, where the dopant metal forms a part of the structural framework, often lead to more resilient species. Zeotype frameworks, particularly aluminophosphates (AlPOs), are excellent hosts for this type of substitution pathway. The basic AlPO framework is constructed of alternating AlO_4_ and PO_4_ tetrahedra, joined through corner sharing Al-O-P bonds. These primary building units (PBUs) then combine to form a range of secondary building units (SBUs), which are typically based on combinations of 4 and 6 membered rings. The type and binding motifs of these SBUs then leads to the formation of a specific microporous framework, with pore dimensions ranging from 3 to 8 Å (Pastore et al., 2005).

By substituting framework Al^3+^ or P^5+^ species with dopant metals it is possible to engineer a range of active sites. Redox active sites are created by substituting aluminum with a M^2+/3+^ species, such as cobalt, iron, manganese etc., this allows the metal to alternate between the adjacent available oxidation states, creating the redox species (Frache et al., 2003; Beale et al., 2005). Solid-acid sites, can be introduced into an AlPO framework facilely, but more importantly, the nature and choice of dopant (mono- or multi-), can advantageously modulate the acid strength of the resulting catalyst (Saadoune et al., 2003; Dai et al., 2013; Potter et al., 2013, 2018b; Gianotti et al., 2014; Mortén et al., 2018). This is achieved by deliberately creating a charge imbalance in the framework, such as substitution Al^3+^ with a divalent species such as magnesium or nickel (Saadoune et al., 2003; Mortén et al., 2018), or substituting P^5+^ with a tetravalent species such as Si^4+^ or Ti^4+^ (Figure S1) (Dai et al., 2013; Mortén et al., 2018; Potter et al., 2018b). Acid characteristics of the different species depend on many variables including the size and electronegativity of the metal, the precise substitution mechanism and the framework topology of the AlPO structure (Corà et al., 2003). In our previous work, we show the inclusion of multiple dopant sites is also a viable technique to control the acidity of metal-substituted aluminophosphates (Potter et al., 2013; Gianotti et al., 2014). This led to the synthesis of a novel Mg^2+^Si^4+^AlPO-5 catalyst, which outperformed the analogous mono-dopant Mg^2+^AlPO-5 and Si^4+^AlPO-5 for the both alkylation of benzene, and the Beckmann rearrangement of cyclohexanone oxime, despite the reactions requiring differing acid strengths (Potter et al., 2013; Gianotti et al., 2014). The findings from this study were instrumental in the predictive design of solid catalysts for the acid catalyzed dehydration of ethanol (Potter et al., 2014, 2018a), where we have shown that SiAlPO-34 is a promising catalyst for converting ethanol to ethylene at low (<250°C) temperatures. This is partially attributed to the isolated silicon sites creating effective acid centers, but also the constricting micropores of SiAlPO-34 (3.8 Å), that promote the formation of ethylene over the larger diethyl ether intermediate. In principle, it is possible to keep increasing the amount of Si in the synthesis gel to enhance the concentration of active sites. However, in our previous work (Potter et al., 2017) we have shown that increasing the Si quantity leads to type III substitution and Si islanding, lowering the overall number of acid sites. We have therefore decided instead to keep the Si loading constant, relative to our SAPO-34 procedure, and instead add a second dopant. To probe the mechanism of the acid-catalyzed process we required a metal with limited redox capability, that would undergo type I substitution, to not compete with the Si for phosphorus substitution (type II). Mg is known to produce stronger Brønsted acid sites when inserted into an AlPO framework (Potter et al., 2013; Gianotti et al., 2014), therefore allowing us to probe the influence of additional stronger acid sites on our catalytic pathway. As such, MgSiAlPO-34 was chosen, as one can control the isomorphous substitution of Mg(II) sites in framework positions of Al(III) sites via a type 1 substitution mechanism, yielding isolated active sites for probing the influence of stronger acid sites on the kinetic pathway of ethanol dehydration.

Zeolites have also been widely used in the dehydration of ethanol to ethylene (Phung et al., 2015; Kadam and Shamzhy, 2018; Li X. et al., 2018; Masih et al., 2019), facing similar challenges of selectively forming ethylene at lower temperatures. It has been shown that zeolites preferentially form diethyl ether at lower temperatures, and that ethylene formation is only favored above 215°C (Kadam and Shamzhy, 2018). Though H-FER and H-USY can achieve high ethylene yields at 300°C, however similar systems are hampered by the formation of longer-chain by-products, leading to coking (Phung et al., 2015; Li X. et al., 2018). In our previous work with SAPO-34 we did not see any products aside from diethyl ether and ethylene, suggesting that the smaller pore may play a significant role in ethylene formation (Potter et al., 2014, 2018a). The benefits of smaller pores have been investigated by others, comparing RHO and MFI zeolites, where the smaller pore of RHO lead to superior ethylene selectivity, alongside a higher quantity of medium-strong acid sites (Masih et al., 2019). As such, we discuss the design of the multi-dopant MgSiAPO-34 framework, and the effect the inclusion of magnesium has on the resulting acid strength, catalytic performance, and reactor design through computational fluid dynamics (CFD) simulations.

Various forms of MgSiAlPO-34 have previously been synthesized (Zhang et al., 2008; Salmasi et al., 2011; Wang et al., 2017; Abdulkadir et al., 2019), with particular emphasis on the methanol-to-olefin (MTO) reaction, where SiAlPO-34 has been the industrial standard for many decades. Work by Salmasi et al. showed that adding magnesium to the SiAlPO-34 framework reduced the total number of acid sites, but resulted in a greater proportion of “strong” acid sites (Salmasi et al., 2011). This led to superior catalytic performance over a longer time period, extending the lifetime of the system. This finding was counter-intuitive, as framework substituted magnesium typically creates stronger acid sites, and therefore the above finding could result from the formation of extra-framework magnesium sites (Salmasi et al., 2011). The latter is evidenced from reports on varying the magnesium content of the SiAlPO-34 species (Zhang et al., 2008), where initially small amounts of magnesium in the framework (0.33 wt%) result in increased overall acidity. However, higher loadings (0.83 and 1.65 wt%) significantly decreases the acidity to 85 and 58 % (respectively) of the original SiAlPO-34 system. It was however shown that, with the exception of the highest loading of magnesium (1.65 wt%), the other catalysts resulted in improved activity for the conversion of chloromethane to C_2_-C_3_ hydrocarbons. We therefore intend to see the influence of incorporating small quantities of Mg^2+^ ions into the framework of SiAlPO-34, using a unique synthesis procedure, to promote isomorphous substitution of Mg^2+^ and Si^4+^ ions, as single-site entities. In line with our previous work, we have carried out in-depth kinetic analysis of solid acid catalyzed dehydration of ethanol to ethylene, as a function of time and temperature, to directly probe the effect of adding magnesium to the framework (Potter et al., 2018a). We will then use these findings as an input for the experimentally defined CFD simulations, to explore local variations in the chemical concentrations across the catalyst bed, with the intention of simultaneously optimizing catalyst and reactor design (Potter et al., 2018a).

## Confirming the Structural Integrity of MgSiAlPO-34

**Figure 2:**
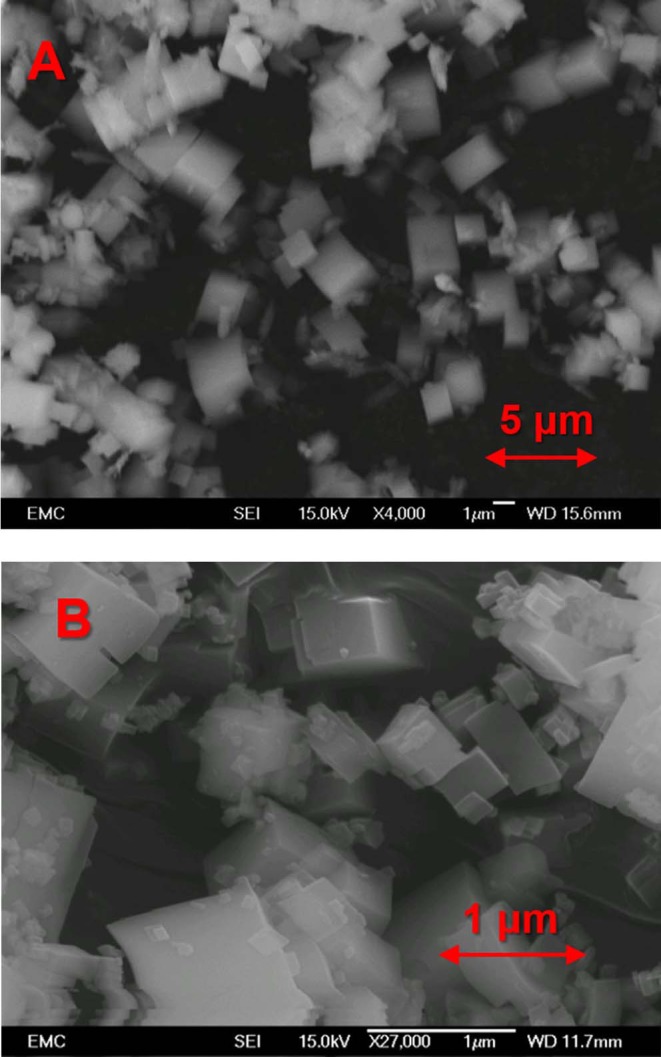
SEM images of **(A)** MgSAPO-34 and **(B)** SAPO-34, both showing predominantly cubic crystals of 1–2 μm in size in both cases.

In our previous work (Potter et al., 2014, 2018a) we have developed synthesis methods to create a phase-pure crystalline SiAlPO-34 catalysts, utilizing tetraethylammonium hydroxide as the structure directing (templating) agent. To synthesize MgSiAlPO-34 we modified this protocol to incorporate a small fraction of magnesium (molar ratio Mg:Si = 1:15), with the aim of limiting extra-framework Mg, promoting isomorphous substitution. This represents the first case (to our knowledge) of MgSiAlPO-34 being synthesized in the absence of triethylamine or morpholine, with all previous reports utilizing either of these templates. The result of our unique multi-dopant synthesis protocol was characterized using a range of physicochemical and *in situ* spectroscopy techniques to confirm the structural integrity of the catalyst, and to explore the influence of magnesium on the acidic properties. Powder X-ray diffraction (XRD) confirmed that our MgSiAlPO-34 catalyst exclusively contains chabazite (CHA) (Figure S2) (Wragg et al., 2012), as expected for the SiAlPO-34 framework, with no visible signs of extra-framework MgO, or any other crystalline phases. On performing a Reitveld refinement (Table S1), the unit cell parameters show excellent agreement with our analogous SiAlPO-34 species, which further confirms phase purity. N_2_ physisorption experiments were used to probe the porosity of the system, and in combination with the XRD findings, confirmed the microporous nature of the system (Table S2), in agreement with the SiAlPO-34 (Sun et al., 2014). To investigate the crystallinity of the system scanning electron microscopy (SEM) was used to explore the particle morphology, showing smooth cubic crystals of around 1-2 μm in length (Figure 2). Again this is in good agreement with previous observations (Potter et al., 2014, 2018a). ICP analysis (Table S3) shows similar levels of Al, P and Si in the MgSiAlPO-34 and SiAlPO-34 catalysts, as variations are within experimental error. We were however successful in incorporating only a small amount of magnesium into the MgSiAlPO-34 framework, as intended, and lower than any previous studies. The combination of these findings suggests there are very little physicochemical differences on introducing magnesium to the framework. Therefore, we can attribute any changes in acidity or catalytic activity to the nature of the active site.

## Influence of Magnesium on Framework Atoms

**Figure 3:**
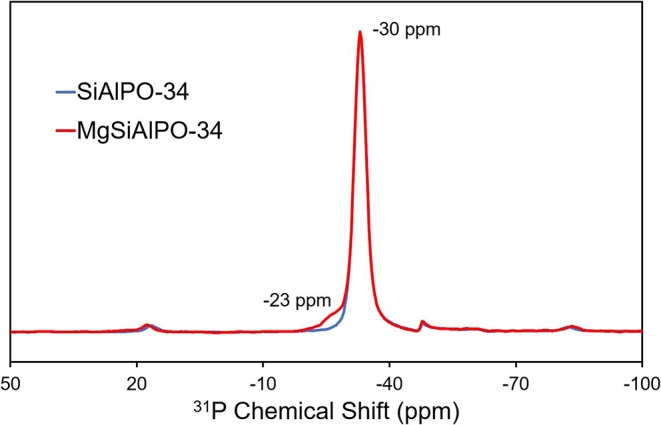
Solid state ^31^P NMR showing the influence of Mg^2+^ incorporation.

The local environment of the framework elements; aluminum, phosphorus and silicon, can be probed using ^27^Al, ^31^P and ^29^Si solid state NMR (respectively). Due to the low loading of magnesium (0.12 wt%), we did not explore the magnesium environment by ssNMR; furthermore, ^25^Mg has a very low sensitivity for NMR, poor natural abundance and quadrupolar. ^27^Al of MgSiAlPO-34 (Figure S3A) shows a peak at 33 ppm, attributed to a Al(OP)_4_ species, with peak shape and position in excellent agreement with SiAlPO-34 (Buchholz et al., 2003). Subtle differences between the spectra occur in the 10–12 ppm region, which is typically attributed to surface alumina sites, bound to water or templating agents (Buchholz et al., 2003). Here we can see MgSiAlPO-34 shows a paucity of these sites, suggesting a slightly more crystalline framework. Probing the ^31^P nuclei (Figure 3) shows a near identical P(OAl)_4_ species at −30 ppm (Buchholz et al., 2003), again showing a nearly identical peak shape to SiAlPO-34, suggesting that the inclusion of magnesium does not significantly influence this feature. However, MgSiAlPO-34 shows an additional feature at −23 ppm, which has previously been attributed to P(OAl)_3_(OMg) species (Deng et al., 1995; Zhang et al., 2008), suggesting that magnesium has indeed been isomorphously substituted into the framework, occupying an aluminum site *via* type I substitution (Gianotti et al., 2014). Similarly the ^29^Si NMR (Figure S3B) is in excellent agreement between the two catalysts, both show a prominent signal at −95 ppm, attributed to Si(OAl)_4_ environments, suggesting type II substitution (Gianotti et al., 2014) and isolated silicon atoms (Blackwell and Patton, 1988). Again, identical line shape shows the addition of small quantities of magnesium has no significant effect on this feature. Therefore, we conclude that MAS NMR demonstrates that the addition of magnesium has only subtly changed the chemical environments of the framework, with the ^31^P NMR showing the presence of framework-substituted magnesium ions (Figure 3).

**Figure 4:**
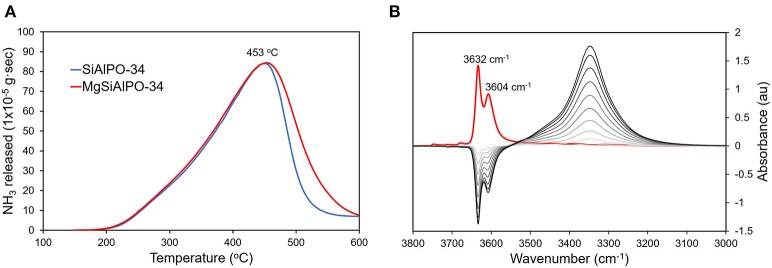
**(A)** NH_3_-Temperature Programmed Desorption (TPD) data comparing the total number, and relative strength of acid sites in MgSAPO-34 and SAPO-34 and **(B)** *in situ* FTIR data showing CO absorption on MgSAPO-34.

Ammonia-probed temperature programmed desorption (NH_3_-TPD) was used to explore the influence of magnesium on the acidity of the catalyst. The TPD data for MgSiAlPO-34 and SiAlPO-34 show near identical behavior up until 450°C (Figure 4A). This suggests that the weaker acid sites, attributed to framework silicon and surface hydroxyl groups, are unaffected by magnesium. Above 450°C, MgSiAlPO-34 shows notably more stronger acid sites, whereas SiAlPO-34 shows a steep decline, indicating fewer stronger acid sites. Throughout our discussion we carefully use the word “stronger” to describe the Mg acid sites. As although these acid sites are among the strongest one can engineer into an AlPO framework, they are still notably weaker than those in zeolites and other solid acid catalysts. Quantifying the area under these signal (Tables S4, S5) shows that MgSiAlPO-34 has significantly more acid sites than SiAlPO-34 (0.944 and 0.822 mmol/g, respectively). We note that the small differences in silicon loading (SiAlPO-34 3.4 wt%, MgSiAlPO-34 3.6 wt%, Table S3) is not significant to account for the difference in acid sites measured by NH_3_-TPD, further inferring that the incorporation of magnesium has a notable influence. We note that from ICP analysis, SiAlPO-34 should theoretically have 1.211 mmol/g of acid sites (based on Si loading), and MgSiAlPO-34 should have 1.335 mmol/g of acid sites (on the basis of Mg + Si loading), which is higher than the values detected by NH_3_-TPD (Table S6). As the ^29^Si NMR revealed the presence of isolated silicon species, we believe that the discrepancy between the theoretical and experimental NH_3_-TPD values must arise from pore-blockage. As SiAlPO-34 is a small-pored framework (3.8 Å), then it is conceivable that bound NH_3_ species could block the pores, hindering access to other available sites. We also note that MgSiAlPO-34 has a greater number of stronger acid sites (>450°C) than SiAlPO-34, and also a greater proportion of stronger acid sites (by 15%). This is in good agreement with previous findings (Zhang et al., 2008), who also showed the total acidity would increase, when Mg loadings below 0.33 wt% were included in the SAPO-34 catalyst. As our Mg loading is 0.11 wt% (Table S3), our results are in good agreement with these findings. Overall this suggests that substituting magnesium into the framework results in the formation of stronger acid sites, in accordance with previous experimental and computational findings (Corà et al., 2003; Potter et al., 2013; Gianotti et al., 2014).

FT-IR experiments focussing on the hydroxyl region (3800 – 3000 cm^−1^) of dry MgSiAlPO-34 reveal analogous characteristics toSiAlPO-34 (Figures S4A,B), showing two strong hydroxyl features at 3,632 and 3,604 cm^−1^, which can be attributed to Brønsted acid sites from silicon framework substitution, giving Al-OH-Si species (Figure S4A) (Smith et al., 1996; Bordiga et al., 2005; Martins et al., 2007). The peak is split due to the two different OH positions, with protons residing in either the 6 or the 6-6 SBUs (Smith et al., 1996; Bordiga et al., 2005; Martins et al., 2007). We also see the typical P-OH band at around 3,678 cm^−1^ and a band at 3,748 cm^1^, attributed to extra framework Si-OH species, both of which are ubiquitous in SiAlPO materials (Figure S4B) (Smith et al., 1996; Bordiga et al., 2005; Martins et al., 2007). A feature, unique to MgSiAlPO-34, is also present at 3,711 cm^1^, which can be attributed to the presence of magnesium in the system. On dosing MgSiAlPO-34 with CO to collect *in situ* FT-IR data, the peaks at 3,632 and 3,604 cm^1^ completely diminish, showing that protons are able to interact with the CO probes (Figure 4B). The CO binding causes a shift in the frequency of the hydroxyl group, to a lower energy, as seen by the appearance of a feature at 3,343 cm^−1^ (Smith et al., 1996; Bordiga et al., 2005; Martins et al., 2007). In the CO stretching region (2250 – 2100 cm^−1^), two features appear with increasing CO concentrations. The primary feature at 2,172 cm^−1^ is attributed to CO bound to Brønsted acid sites, while the secondary feature at 2,141 cm^−1^ is physisorbed “liquid-like” CO (Figure S4C) (Smith et al., 1996; Bordiga et al., 2005; Martins et al., 2007). Again, this is in excellent agreement with our previous work on SiAlPO-34 (Potter et al., 2018a). Integrating the CO signal gives a value of 1.39 au for MgSiAlPO-34, compared to 1.08 au for SiAlPO-34, confirming that the addition of magnesium increases the number of acid sites, as seen through NH_3_-TPD.

## Catalytic Behavior of MgSiAlPO-34

The efficacy of the multi-dopant substitution in MgSiAlPO-34 was contrasted with the mono-dopant SiAlPO-34, by using the low-temperature, catalytic dehydration of ethanol as a model reaction. The wider benefits of designing catalysts that can operate at low-temperatures, notwithstanding the energy savings, extends scope for deployment of bio-based feedstocks, such as bioethanol that can be derived from sugarcane waste (bagasse) and corn. Bioethanol has been identified as a possible sustainable energy source for the future with developing countries such as Brazil already utilizing a significant amount for fuel, from the fermentation of sugar cane (Hira and Guilherme de Oliveira, 2009). Extending this notion it is possible to also use bioethanol as a feedstock for bulk and fine chemical production Hira and Guilherme de Oliveira (2009) reducing the requirements for crude oil. Ethylene is used globally as a plastic and pharmaceutical precursor, the vast majority coming from steam cracking (Zhang and Yu, 2013), and low-temperature dehydration of bioethanol could offer a sustainable solution for ethylene production.

**Figure 5:**
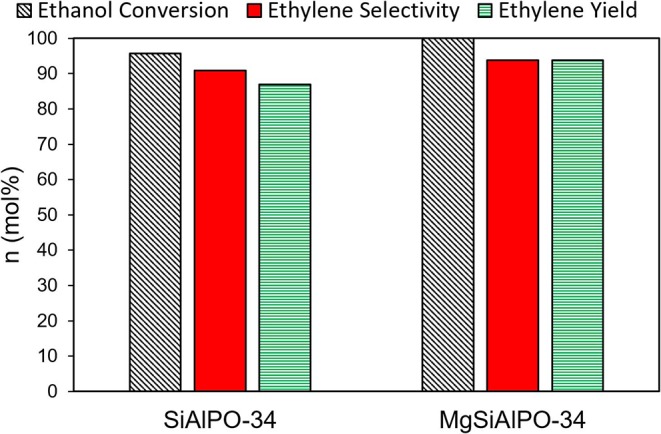
Comparing the ethylene production of SAPO-34 and MgSAPO-34 catalysts at 225°C, 0.3 g catalyst, He carrier gas = 25 mL/min, WHSV = 0.3 hr^−1^.

Under identical reactions conditions, MgSiAlPO-34 achieves an overall ethylene yield of 94 mol%, compared to 87 mol% for SiAlPO-34 (Figure 5 and Table S7), highlighting the benefits of our design strategy to form a multi-dopant catalyst. The improved catalytic behavior is likely due to the addition of “stronger” acid sites from magnesium doped into the framework. As we saw no other products, we can conclude that these Mg acid sites were not sufficiently strong enough to enforce unwanted side reactions, such as ethylene polymerisation, and are therefore more favorable than those present in zeolites. In order to better understand the influence of magnesium, a kinetic study was performed, varying contact time, and temperature, to contrast with previous work on SiAlPO-34. We also show that the MgSiAlPO-34 maintains a high level of activity after 7 h on stream (Figure S5), analogous to SiAlPO-34 in our previous work (Potter et al., 2018a), vindicating the stability of our catalyst.

**Figure 6:**
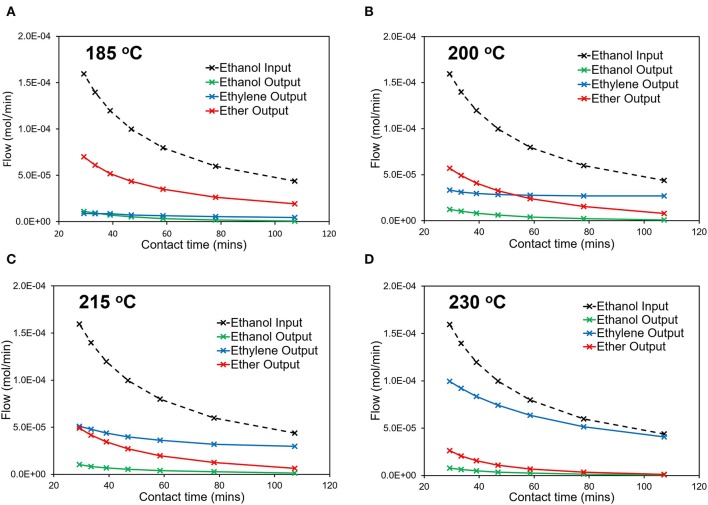
Catalytic data detailing how the output stream composition varies with contact time at **(A)** 185°C, **(B)** 200^o^C, **(C)** 215^o^C, and **(D)** 230°C.

Varying ethanol contact time with the multi-dopant catalyst (MgSiAlPO-34), influences the overall reactivity (Figure 6) and, even at the lowest temperature (185°C), a significant amount of ethanol is converted (Figure 6A), primarily forming the intermediate, diethyl ether. The flows are expressed as mol/min for ease of translating to kinetic and CFD analysis. However, care must be taken, as the ethanol input (mol/min) will not necessarily equal the sum of the output flows, due to two moles of ethanol being required to form one mole of diethyl ether. In doing so, this calculation leads to an accurate carbon balance, but cannot always lead to an accurate mole balance. With increased contact times, the ethanol output continues to decrease, suggesting higher conversions at higher contact times. Also while diethyl ether remains the primary product, the relative amount of ethylene increases as contact time increases. This is in line with our previous observations on mono-dopant SiAlPO-34 (Potter et al., 2018a), suggesting at low temperatures the dominant reaction is the formation of diethyl ether. Increasing the temperature, we see a similar trend for the conversion, with minimal ethanol in the output stream, which continues to decreases with increasing contact times. The product distribution also varies, with increasing temperatures, resulting in increased ethylene yields, and lowering diethyl ether formation. To emphasize this point, above 215°C (Figures 6C,D) ethylene becomes the primary product, under our conditions. This is in line with the decomposition of diethyl ether to ethylene, as this is a limiting step in this process (Potter et al., 2018a). Further, the relative amount of ethylene continues to increase as a function of contact time. This is best shown at 200°C (Figure 6B), where the primary product switches from diethyl ether to ethylene in the 40–60 min contact time range. This transition occurs at a lower temperature than SiAlPO-34, where ethylene only becomes the primary product at 215°C (Figure 6C). This suggests that the increased number of stronger acid sites in MgSiAlPO-34, due to the inclusion of magnesium in the framework, is able to promote the formation of the desired ethylene product.

## Kinetics

The rate constants for the three steps were determined in an analogous fashion to our previous work on SiAlPO-34 (Potter et al., 2018a). The product distributions, varying as a function of contact time, were used as inputs to calculate the rate constants of the three steps, at the different temperatures and flow rates. The open-source software Copasi (Hopps et al., 2006) was used to calculate the rate constants for all three steps (Figure S6). We present the individual rate constants established using the multi-set data for the different experimental cases. As per our previous work (Potter et al., 2018a), the individual cases were considered to ascertain whether any reactions are kinetically limited (constant with varying WHSV) or diffusion limited (varying with WHSV). The present studies show the rate constants for the multi-dopant MgSiAlPO-34 differ from those presented previously for SiAlPO-34. For SiAlPO-34, the rate constants for reactions a and b (k_a_ and k_b_) were roughly constant, regardless of the WHSV, therefore these steps were considered kinetically limited. On the other hand, the rate constants for *step c* (k_c_) decreased with increasing flow, suggesting it was diffusion limited at lower WHSVs. With the MgSiAlPO-34, k_a_, k_b_ and k_c_ vary with increasing WHSV (Figure S6), before converging at higher WHSVs in the range of 0.92–1.47 hr^−1^. This suggests that in the current MgSiAlPO-34 case, the chemical transformations, at low flow rate, are occurring sufficiently fast that the reaction is now limited by diffusion, due to poor mass-transfer. This deviation is most pronounced at highest temperature studied (230°C), as again the kinetic reaction is occurring so rapidly, that the diffusion of reactants and products to the active site, is not the rate determining step. The convergence of the rate constant at higher flows shows the reaction transitions to being chemically limited, likely due to shorter contact time, leading to the formation of fewer ethoxy intermediates. Therefore, our investigation will consider the kinetic rate constant values for the higher WHSVs (0.92–1.47 hr^−1^) only, ensuring we are in the kinetically limited regime, to extract the activation energy and pre-exponential factors *via* an Arrhenius plot (Table 1 and Figures S7, S8). In this region the Arrhenius plot followed a linear trend (Figure S7), yielding ln(A) and E_a_ values in a similar range to those of SiAlPO-34 (Table 1 and Figure S9). Comparing these rate constants as a function of temperature (Figure S8) should be done carefully, as the rate constants have different units due to their different orders; k_a_ being first order (s^−1^) and k_b_ and k_c_ are second order (ml mol^−1^ s^−1^). As such direct comparison is only possible between k_b_ and k_c_, both increase as expected with temperature (Figure S8) however due to the higher activation energy and pre-exponential factor (Table 1) k_b_ increases more drastically with temperature than k_c_. This suggests *step c* (ethylene formation from diethyl ether) is more susceptible to increases in temperature than *step b* (diethyl ether formation).

**Table 1:** Calculated activation energies and pre-exponential factors for the rate constant of the individual reaction steps of MgSiAlPO-34, using the 0.92–1.47 hr^−1^ WHSVs cases.

**Rate constant**	**E_**a**_ (kJ/mol)**	ln(A)^*^
k_a_	93.15	14.569
k_b_	64.37	23.729
k_c_	144.32	41.250

* *A varies in units due to the difference in reaction order of the different reactions, for k_a_ (first order) A has units s^−1^, for k_b_ and k_c_ (both second order, as per our previous work, Potter et al., 2018a), A has units of ml mol^−1^ s^−1^*.

Comparing the Arrhenius plots of MgSiAlPO-34 and SiAlPO-34 (Figures S7–S9) shows the influence of the additional stronger Brønsted acid sites, brought about by the incorporation of Mg^2+^ ions into the framework. For SiAlPO-34, *step a* was found to have little influence on the activity of the system, and in the MgSiAlPO-34 case, we see that the rate constants are even lower (Figure S9A), suggesting this will play even less of a role under the conditions studied. Extending the data points to higher temperatures would see the MgSiAlPO-34 k_a_ surpass that of SiAPO-34, and potentially lead to this pathway becoming more significant, suggesting that stronger acid sites can promote the direct dehydration of ethanol to ethylene under certain conditions (further work in progress and outside the scope of this study). We note that k_b_ shows a significant decrease in activation energy on including Mg^2+^ ions (Figure S9B), with MgSiAlPO-34 having an activation energy of 64.4 kJ/mol, compared to SiAlPO-34 with 70.7 kJ/mol (Potter et al., 2018a). Therefore, we can conclude that the presence of stronger acid sites in the multi-dopant catalyst lowers the energy barrier for the formation of the diethyl ether intermediate. MgSiAlPO-34 showed higher k_b_ values across the whole temperature range studied, and the lower activation energy also confirms that it would be a more suitable candidate for diethyl ether (and ethylene) production at lower temperatures. In terms of k_c_ (Figure S9C), the activation energy of the two species is almost identical, suggesting the enhanced acidity has little influence on the decomposition of diethyl ether to yield ethylene, *step c* (page S4). MgSiAlPO-34 however maintains a higher rate constant than SiAlPO-34, due to a higher pre-exponential factor, suggesting a greater number of collisions between the molecules. This may simply be a product of the greater number of acid sites present in the MgSiAlPO-34 (0.944 mmol/g) compared to SiAPO-34 (0.822 mmol/g), providing more sites to facilitate this reaction, or to a more specific interplay between these sites located a proximal positions within the framework (Potter et al., 2013, 2014, 2018a; Gianotti et al., 2014). However, the similar activation energies suggest that the change in overall acid site strength has little influence on the reaction pathway. Therefore, we conclude the enhanced catalytic activity of the MgSiAlPO-34 over SiAPO-34 is due to the stronger acid sites, generated through multi-dopant substitution, promoting the formation of the diethyl ether intermediate, and the subsequent modulation of Mg^2+^Si^4+^ active species providing more sites to form ethylene form the diethyl ether.

## Computational Fluid Dynamics of the MgSiAlPO-34 System

Two-dimensional CFD simulations were performed using a reactive porous model in ANSYS Fluent 17.1^1^. The model set up is described in detail in our previous work (Potter et al., 2018a). We extend this study to the reaction kinetics of the multi-dopant MgSiAlPO-34 experiment, presented in Table 1; focusing on the presence of Mg^2+^ and formulating additional active sites. Comparing the simulated and experimental mole fractions over a range of temperatures for the 0.92 hr^−1^ and 1.42 hr^−1^ WHSV, (Figure 6), shows the simulated results capture the key profiles of the existing products, although some subtle deviations occur at 200 and 215°C. This is likely due to the rate constants at these temperatures deviating more from the linear trend for each of the reactions (Figure S6). As noted previously for the SiAlPO-34 case, it is between these two temperatures that a transition is observed, where ethylene becomes the dominating product, as opposed to diethyl ether. This transition point is consistent for both the 0.92 and 1.47 hr^−1^ WHSV cases.

**Figure 7:**
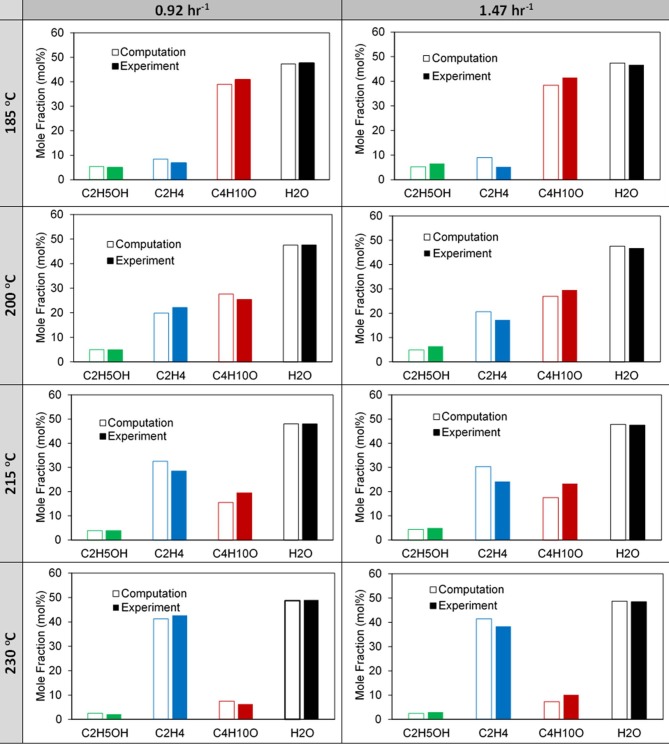
Comparison of computational and experimental exiting mole fractions for the 0.92 and 1.47 h^−1^ WHSV cases for increasing reactor temperature.

**Figure 8:**
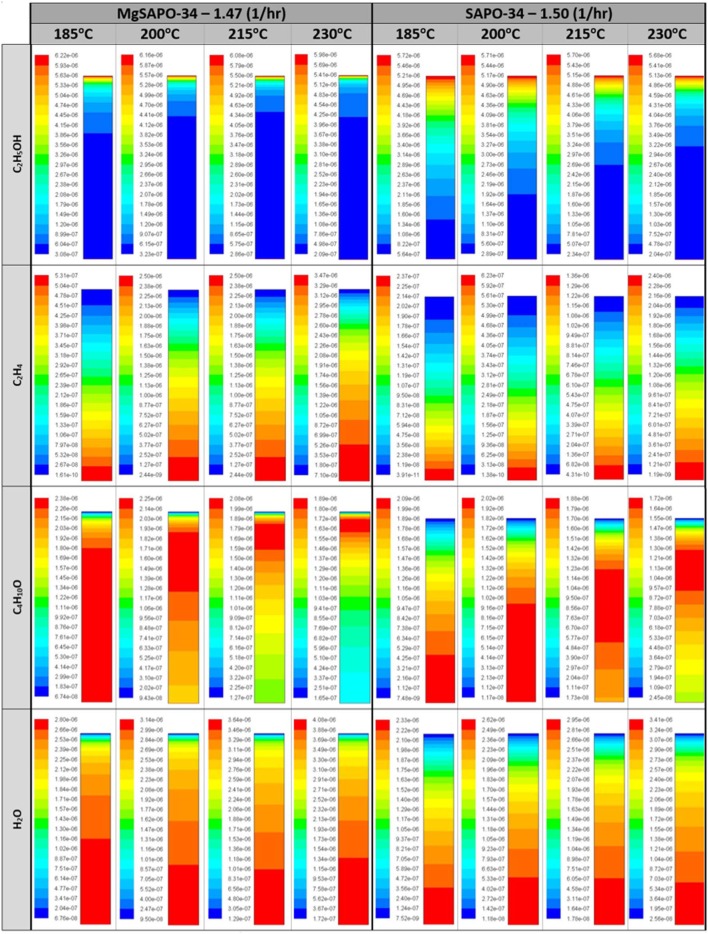
Molar concentration (mol/ml) distribution of species at varying temperatures for the MgSAPO-34 at 1.47 WHSV (hr^−1^) and the SAPO-34 at 1.5 WHSV (hr^−1^) with varying reactor height (y-axis), as the chemicals are introduced at the top of the reactor and exit through the bottom.

The computationally predicted outlet stream concentrations and mole fractions, from our CFD model, showed excellent agreement with the experimental values (Figure 7 and Table S8) over a range of temperatures and WHSV values. As such we are confident in the models ability to replicate the experimental values, thus validating it. Following this we then observed the spatial variation of the reaction components within the catalytic bed of the reactor, similar to our previous work (Figure 8) (Potter et al., 2018a). Comparing MgSiAlPO-34 and SiAlPO-34 under similar conditions (WHSV of 1.47 and 1.5 hr^−1^, respectively), further emphasizes the influence of the additional stronger acid sites, present in the multi-dopant catalyst. MgSiAlPO-34 is able to more readily activate ethanol than SiAPO-34, due to the faster decline in ethanol concentration down the catalytic bed, across all temperatures. This is in good agreement with the higher k_b_ values in the MgSiAlPO-34 kinetic analysis (Table 1), and as a result, means diethyl ether reaches a maximum concentration much earlier in the catalytic bed. It is envisaged the presence of the multi-dopant active sites and, possibly their proximal location within the framework architecture, accelerates the overall rate of the reaction, due to the stronger acidity of this modulated catalyst. As the formation of ethylene from diethyl ether is second order, with respect to diethyl ether, then increased diethyl ether concentration will subsequently increase the formation of ethylene in the catalytic reaction. As such, noticeably more ethylene is produced in the reaction, while reaching a maximum value earlier in the bed, compared to the SiAlPO-34 case. The latter observation suggests that the catalytic bed could even be shortened, which on larger scales would result in significant reductions in cost of catalyst, or allow the temperature to be decreased further, offering additional process benefits.

## Conclusion

By utilizing a novel synthesis protocol with tetraethylammonium hydroxide we were able to form phase-pure, crystalline MgSiAlPO-34. Through a range of physicochemical characterization procedures the structural and compositional integrity were evaluated, with solid state NMR suggesting the isomorphous substitution of Mg^2+^ for Al^3+^ via type I substitution. Despite structural similarities (with the mono-dopant SiAlPO-34), incorporating both Mg^2+^ and Si^4+^ ions simultaneously into the multi-dopant MgSiAlPO-34 chabazite framework, altered the acidic characteristics of the catalytic system. This prompted an increase in both the quantity and relative strength of the Brønsted acid sites, compared to mono-substituted SiAlPO-34. These differences in acidity initially showed that MgSiAlPO-34 was a superior catalyst for ethanol dehydration, producing improved ethylene yields under analogous conditions to SiAlPO-34. Further kinetic and CFD work on the system highlights that this improvement is due to two factors. First the stronger acid sites lower the energy barrier for the formation of the diethyl ether intermediate, thereby increasing the rate of reaction for subsequent ethylene formation. Furthermore, the increased number of solid-acid sites, possibly facilitated through proximal location of the Mg^2+^ and Si^4+^ species, facilitates more collisions for the latter step, also leading to greater ethylene yields. In line with these findings, CFD shows diethyl ether reaches a maximum concentration much higher up the catalyst bed in MgSiAlPO-34 than SiAlPO-34, facilitating the improved ethylene yields. Overall this work reinforces the benefits of multi-dopant substitution in framework architectures, which lead to improved product yields, under less energy intensive reaction conditions, furthering the need for unique and novel synthetic methods for such systems.

## Data Availability Statement

All datasets generated for this study are included in the article/Supplementary Material.

## Author Contributions

MP performed catalyst synthesis, physicochemical characterization, and catalyst testing. L-MA performed the CFD modeling and kinetic analysis. MC performed NMR characterization and data analysis. TM performed TPD and FTIR experiments and data analysis. RR assisted with initial reactor design and associated theories in this paper.

### Conflict of Interest

TM was employed by the company UOP, A Honeywell company. The remaining authors declare that the research was conducted in the absence of any commercial or financial relationships that could be construed as a potential conflict of interest.

## Footnotes

^1^ANSYS Fluent 17.1, http://www.ansys.com/ (accessed December 2017).
