# Supplementary material for: Designing Multi-Dopant Species in Microporous Architectures to Probe Reaction Pathways in Solid-Acid Catalysis
Source: Front Chem. 2020 Mar 17;8:171. doi: 10.3389/fchem.2020.00171 (PMC7089933; doi:10.3389/fchem.2020.00171)
Supplement: Supplementary file 1 [file Data_Sheet_1.PDF]

SUPPLEMENTARY INFORMATION

Designing multi-dopant species in microporous architectures to probe reaction pathways in solid-acid catalysis

Matthew E. Potter[1]*, Lindsay-Marie Armstrong[1]*, Marina Carravetta[1], Thomas M. Mezza[2] and Robert Raja[1]

1) University of Southampton, University Road, Highfield, Southampton, SO17 1BJ, UK.

2) UOP, a Honeywell company, Des Plaines, IL 60017, USA.

Invited Paper

*MEP = M.E.Potter@soton.ac.uk, LMA = L.Armstrong@soton.ac.uk

TABLE OF CONTENTS

**Title**	**Page No.**
Experimental details	S2
Catalyst synthesis	S2
Physicochemical characterisation	S2
MAS NMR	S2
Acid-site characterisation	S3
Catalysis procedure	S3
Kinetic analysis	S4
Computational fluid dynamics simulations	S4
AlPO substitution schematic	S5
Physicochemical characterisation data	S5
Solid state NMR spectra	S6
NH_3-TPD analysis	S7
FTIR spectra	S8
Catalytic data	S9
Kinetic analysis	S10
Computational fluid dynamics data	S13
References	S14

Experimental details

Catalyst synthesis

MgSiAlPO-34 samples were prepared by the following procedure: 15.1 g aluminium isopropoxide was mixed with 31.1 g tetraethylammonium hydroxide solution (35% in water) and stirred for about 1.5 h. Then, 0.66 g of fumed silica was added to the mixture under vigorous stirring. Magnesium acetate tetrahydrate was then dissolved in 3 g of water and added dropwise to the gel. Finally, 8.52 g of phosphoric acid diluted in 9.2 g of water was added drop wise to this mixture. Stirring continued for another 2 h. The final gel composition was:

$$0.02\ Mg/Al_2O_3/P_2O_5/0.30\ SiO_2/2.0\ TEAOH/50\ H_2O$$

Hydrothermal crystallization was performed at 200 ^oC for 60 h in a steel autoclave. After crystallization, the product was separated by centrifugation and washed several times with water. The product was then dried at 100 ^oC overnight. The sample was calcined under a flow of air for 16 hours at 600 ^oC, with a heating ramp of 2.5 ^oC/min.

SiAlPO-34 was synthesized and characterized as per our previous work.[S1]

Physicochemical characterization

ICP-OES measurements were performed by Medac.

Phase purity and crystallinity of materials was confirmed by powder X-ray diffraction. Powder X-ray diffraction (PXD) was performed on a Bruker D2 Phaser diffractometer with Cu Kα1 radiation. Patterns were run over a 2θ range of 5 – 40 ^{o} with a scan speed of 3 ^{o}/min and an increment of 0.02 ^{o}.

Scanning electron microscopy was carried out using a Jeol JSM-5910, and samples were prepared by Au sputtering prior to imaging.

BET surface area measurements were performed at 77 K, on a sample dried under 20 mTorr of vacuum at 120 ^oC overnight. Analysis was performed on a Micromeritics Gemini 2375 surface area analyzer. Surface area was calculated using the BET model.

MAS NMR

All NMR measurements were performed on a Chemagnetics Infinity 400 spectrometer on a 4 mm MAS double-resonance APEX probe. For all samples, approximately 100 mg of material was quickly transferred in a thin wall zirconium oxide rotor and then spun at 8 kHz using compressed nitrogen, in order to prevent sample degradation in air, for bearing, drive and purge. The nitrogen gas was generated in-house from evaporation of liquid nitrogen in high pressure 1300 L tanks suitably connected to the NMR facility. ^{27}Al NMR experiments were performed using direct acquisition (128 scans with and a pulse delay of 2 s between scans). ^{31}P NMR data were acquired with direct acquisition (4 scans and 120 s delay between scans). ^{29}Si NMR data for all 1D experiments were performed using cross-polarization and SPINAL64 decoupling.[S1] Typical spectra were acquired with 8192 scans and 2 s between scans. The

chemical shift axes in the ^{27}Al, ^{31}P and ^{29}Si spectra were referenced using 1M $AlCl_3$ aqueous solution (0 ppm), 85 % H_3PO_4 (0 ppm) and silicon rubber (–22.42 ppm) respectively. The NMR data was processed using matNMR.

Acid site characterization

CO-probed FT-IR experiments were performed in a custom designed IR flow cell that allowed for sample heating and cryogenic cooling. Freshly calcined samples were ground and pressed into 13 mm diameter self-supporting pellets (~8 mg/cm^2) and heated at 10 ^{o}C/min to 550 ^{o}C in a mixture of 20 % O_2 in N_2 [Matheson UHP grade further purified using a P400 air purifier(VICI)] and held for 1 h. The flow was then switched to helium [Matheson UHP grade further purified using a P-100 helium purifier(VICI) and an indicating OMI-1 purifier(Supelco)] and held for an additional hour. The system was then cooled to ~-175 ^{o}C and a spectrum recorded. Nine 0.02 cm^3 injections of CO (Matheson research purity) were added to the system followed by a final injection of 0.20 cm^3. After each injection, the system was equilibrated for 3 min and a spectrum recorded.

TPD measurements were performed on a custom built system using TCD detectors to monitor ammonia concentration. As-synthesized materials were pretreated by heating at 10 ^{o}C/min to 550 ^{o}C in a 20 % O_2/Helium mixture [Matheson UHP grade passed through a Drierite/molecular sieve gas purifier (Alltech Associates)] and held for 2 h. The samples were exposed to ammonia and allowed to equilibrate at 150 ^{o}C for 8 h. Desorption was performed in flowing helium [Matheson UHP grade further purified with an Oxy-Trap (Alltech Associates) and an indicating OMI-1 purifier (Supelco)] at 10 ^{o}C/min to 600 ^{o}C and held for 40 minutes at 600 ^{o}C.

Catalysis procedure

Catalysis was performed using a custom build flow reactor provided by Cambridge Reactor Design. The reactor comprised of a syringe pump, laptop computer, two mass flow controllers, and reactor with heater and control box. A 224 mm quartz reactor tube (4 mm id, 6 mm od) with a 4 mm high frit 80 mm from the base of the tube and a gas inlet 25.8 mm from the top was placed inside the heater jacket. Liquid (90% ethanol, 10% heptane, latter for GC calibration) and gas flows (helium) were controlled using a Harvard Apparatus Model 33 MA1-55-3333 syringe pump and Brooks IOM585OS mass flow controller respectively and flow rates were input via computer interface.

The output was vaporized and 5 μl samples were injected as a gas into a Varian Star 3400CX gas chromatogram with flame ionization detector (FID). Samples were injected into a Perkin Elmer a HP1 cross linked methylsiloxane (30 m x 0.32 mm x 1 μm film thickness) column. All results shown are the average of two consistent samples.

Conversion was calculated as:

$$100\ \% \times [mol(Ethanol)_{inlet} - mol(Ethanol)_{outlet}]/mol(Ethanol)_{inlet}$$

Ethylene Selectivity was calculated as:

100 % x mol(Ethylene)$_{outlet}$/[mol(Ethylene)$_{outlet}$ + mol(Diethyl ether)$_{outlet}$]

Diethyl ether Selectivity was calculated as:

100 % x mol(Diethyl ether)$_{outlet}$/[mol(Ethylene)$_{outlet}$ + mol(Diethyl ether)$_{outlet}$]

Yields are measured as:

Conversion x Selectivity / 100%

Kinetic analysis

Rate constants were derived using the following reaction steps, as per our previous work:

A) Ethanol → Ethylene + H_2O
B) 2Ethanol → Diethyl Ether + H_2O
C) Diethyl Ether → Ethylene + Ethanol

The Copasi software[S2] takes the concentrations of the chemical species as a function of time, and the expected chemical equations, as an input. The rate of each reaction was given by a kinetic rate law (of order 1, 2 and 2 for reactions a, b and c, respectively,[S3,S4] as described in our previous work,[S5] and the kinetic rate constants were established using a parameter estimation which models the three different proposed reactions simultaneously, with inputs from all data sets from the different operating temperatures and flow rates, and minimizes the value distances between the model and experimental data. We initially applied a genetic algorithm before performing the local optimisation by Levenberg–Marquardt, which is a least-squares approach to solving non-linear problems.

Computational Fluid Dynamics simulations

The cylindrical catalytic bed of the reactor (4 mm diameter and 25 mm height) was discretized into 40000 quadrilateral cells. The investigation into contact time considered temperatures that aligned with the experimental conditions of 185, 200, 215 and 230 ^{o}C; and mass flow rates of ethanol, heptane and helium for a range of WHSV from 0.4 to 1.47 hr^{-1}. A constant wall temperature was set corresponding to the required temperature to align with the experimental conditions. The walls were defined as no-slip boundary conditions. The model accounts for temperature variation due to endo- and exothermic reactions. The temperature of the inlet gases entering was set to the corresponding temperature of the reactor bed, which was fixed for that case. The outlet was defined as an outflow boundary conditions. The inlet and outlet boundaries were set to atmospheric pressure to align with the experimental set up. As with our previous study the CFD reactive porous model is expanded using user-defined functions (UDFs) to incorporate a range of a parameters that are either fundamental constants, or derived experimentally[S5] into the transport equations. The catalytic loading was calculated based on the MgSiAlPO-34 surface-to- volume ratio from the experiments, therefore accounting for the area a reaction can take place in, for each cell, by multiplying the volume of the discretized cell by this ratio. This value was calculated to be 1.76 × 109 m^{-1} (surface area 580 m^2/g, total pore

volume 0.33 cm^3/g). The average pellet diameter (1 µm) was estimated from the SEM images, and a bed porosity of epsilon = 0.4 was used to account for a randomized distribution within the bed of roughly cubic shaped pellets. A flow resistance was applied to the pelletized catalytic region which accounts for the viscous and inertial losses experienced within the packed pellet bed. These parameters were determined according to the semi-empirical Ergun equation for pressure drops within packed beds.[S6] The full model description can be found in our previous works.[S5]

AlPO substitution schematic

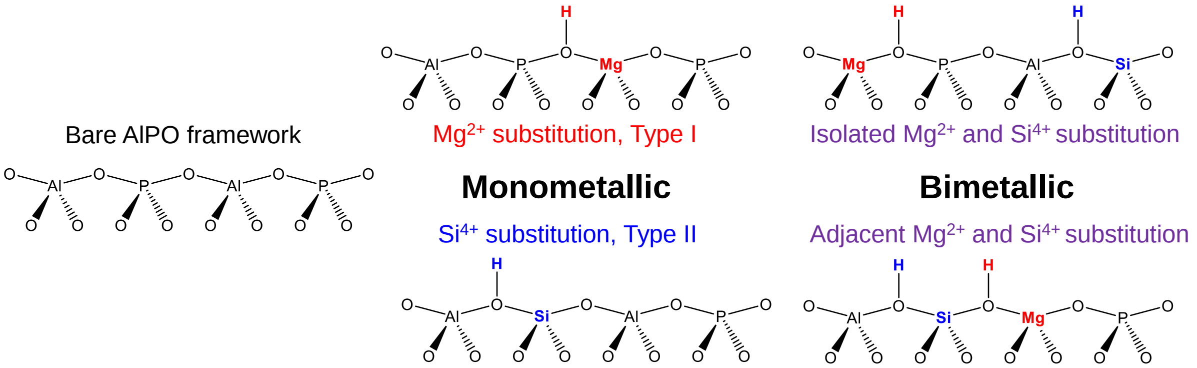

Figure S1: Schematic showing the possible substitution mechanisms in MgSiAlPO-34.

Physicochemical characterisation data

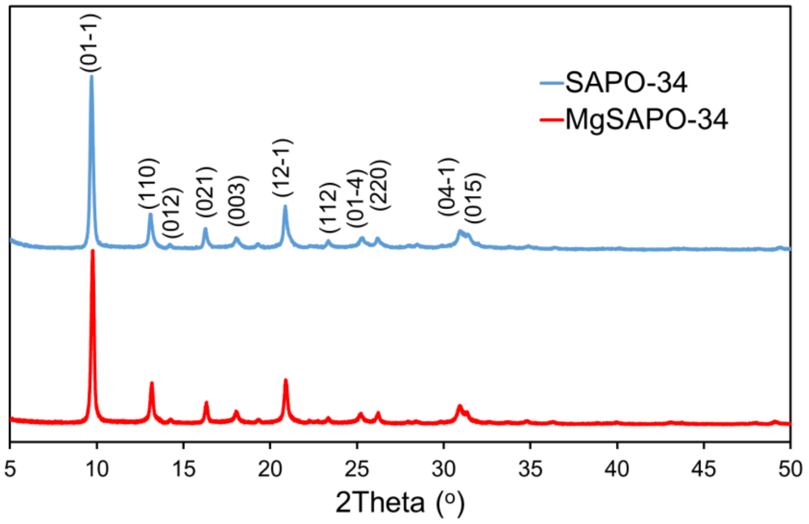

Figure S2: Powder X-ray diffraction patterns showing the phase-purity and crystallinity of MgSiAlPO-34.

Table S1: Unit cell data for SiAlPO-34 and MgSiAlPO-34 as determined using Celref with space group of R-3m.

Catalyst	Alpha (Å)	Gamma (Å)	Volume (Å^3)
SiAlPO-34	13.796	14.983	2469.63
MgSiAlPO-34	13.727	14.810	2416.76

Table S2: Crystallite sizes derived from powder-XRD with Scherrer's equation. Surface area measurements determined using BET.

	SiAlPO-34	MgSiAlPO-34
Crystallite size (nm)	9.46	9.44
BET surface area (m^2/g)	524 ± 13	494 ± 11

Table S3: Inductively coupled plasma measurement results.

	Elemental analysis				
Sample	Al (wt%)	P (wt%)	Si (wt%)	Mg (wt%)	(Mg + Al)/(P + Si) (mol%)
SiAlPO-34	20.6	18.6	2.99	0	1.08
MgSiAlPO-34	18.9	16.3	2.97	0.11	1.12

<u>Solid state NMR spectra</u>

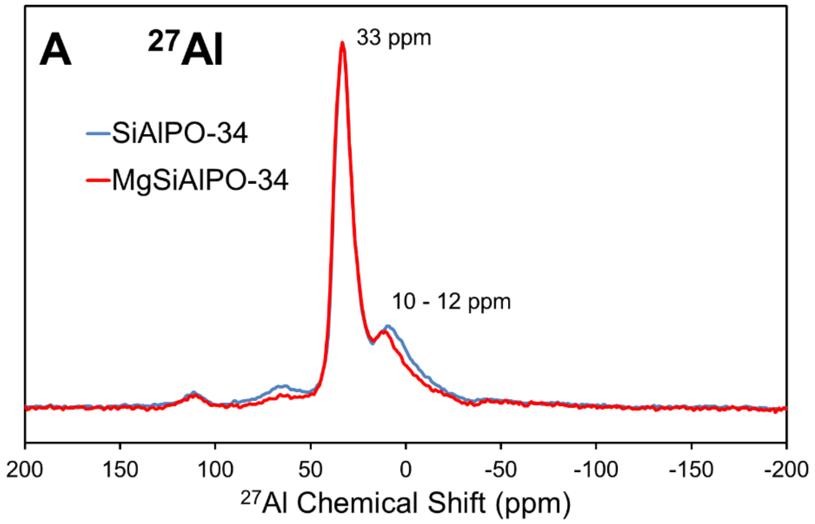

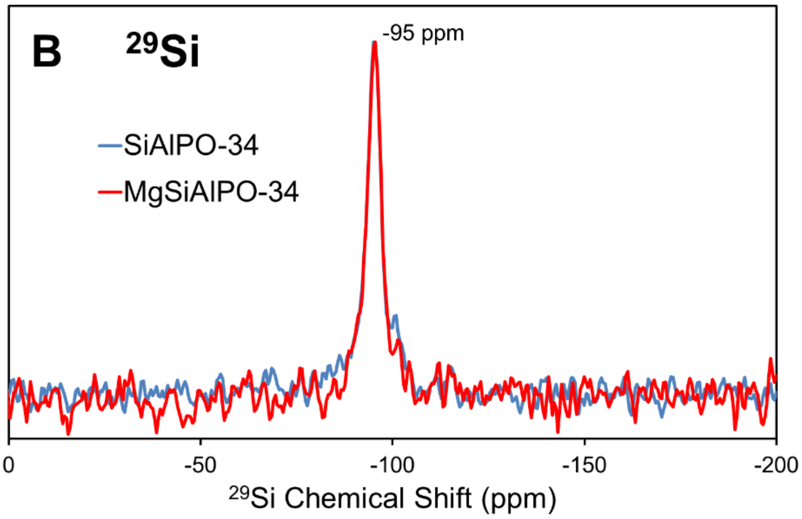

Figure S3: Solid state NMR measurements comparing MgSiAlPO-34 and SiAlPO-34 focussing on the A) ^{27}Al and B) ^{29}Si nuclei.

NH$_3$-TPD analysis

Table S4: NH$_3$-Temperature Programmed Desorption (TPD) summary in mmol/g

Sample	Integration Results (mmol/g)					
	150-200 °C	200-300 °C	300-400 °C	400-500 °C	500-600 °C	Total
SiAlPO-34	0.001	0.060	0.253	0.427	0.081	0.822
MgSiAlPO-34	0.001	0.064	0.264	0.470	0.146	0.944

Table S5: NH$_3$-Temperature Programmed Desorption (TPD) summary as a percentage.

Sample	Integration Results (%)				
	150-200 °C	200-300 °C	300-400 °C	400-500 °C	500-600 °C
SiAlPO-34	0	7	31	52	10
MgSiAlPO-34	0	7	28	50	15

Table S6: Contrasting theoretical acid loading with experimental TPD data.

	SiAlPO-34	MgSiAlPO-34
Si (wt%)	3.4	3.6
Mg (wt%)	0.00	0.13
Si (mmol/g)	1.211	1.282
Mg (mmol/g)	0.000	0.053
Theoretical acid sites (mmol/g)	1.211	1.335
Experimental acid sites (mmol/g)	0.822	0.944

FTIR spectra

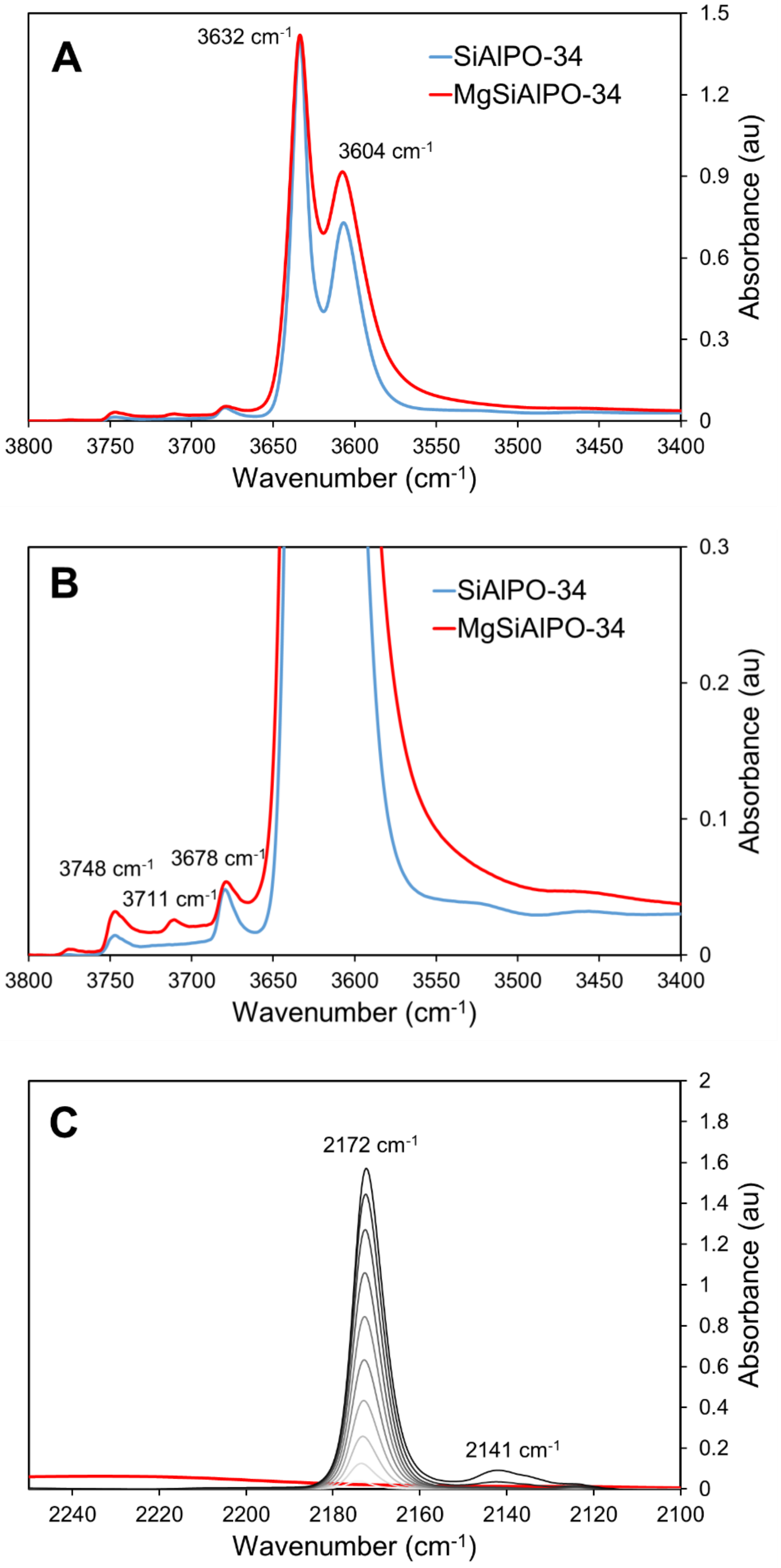

Figure S4: FTIR spectra showing A) The differences between MgSiAlPO-34 and SiAlPO-34 in the hydroxyl region, B) Emphasising the differences in Al-OH, P-OH and Si-OH features

and C) The CO stretches appearing as CO is absorbed onto the MgSiAlPO-34 species. Each grey line represents the spectra following a further injection of 0.2 cm^3 of CO, with subsequent injections being a darker shade. The red line represents the bare sample with no CO present.

Catalytic data

Table S7: Comparing the ethylene production of SiAlPO-34 and MgSiAlPO-34 catalysts with other literature data and commercial processes.

Catalyst	T/°C	WHSV/hr^{-1}	Conv/mol%[a]	Sel/mol%[b]	Ref
		**Commercial Systems**			
Braskem, Syndol	450	0.56	99	97	S7
Solvay Indupa, Syndol	200-400	0.33-0.43	99	97	S8
Lummus, γ-Al_2O_3	400	0.66	99	99	S9
Petrobras, Al_2O_3-SiO_2	300-440	0.84-7.0	99	98	S10
Shanghai Engineering, H-ZSM-5	260	2.4	98	99	S11
		**Academic reports**			
SiAlPO-5	250	4.38	87	31	S12
SiAlPO-34	250	4.38	93	76	S12
TPA	250	12	53	73	S13
MPA	250	12	10	30	S13
STA	250	12	69	64	S13
H-ZSM-5 (30)	300	N/A	90	79	S14
20P-H-ZSM-5 (30)	300	N/A	71	35	S14
Al_2O_3	300	1.43	98	100	S15
SiO_2/Al_2O_3 (5)	300	1.43	74	24	S15
SiO_2	300	1.43	0.6	25	S15
H-FER (20)	200	1.43	70	5	S16
H-MFI (280)	200	1.43	45	1	S16
H-MFI (50)	200	1.43	82	12	S16
H-MOR (20)	200	1.43	92	75	S16
H-BEA (20)	200	1.43	85	11	S16
H-Y (5.1)	200	1.43	58	2	S16
H-USY (30)	200	1.43	63	3	S16
TiO_2/γ-Al_2O_3	360-500	26-234	99	100	S17
H-ZSM-5	275	2.37	99	99	S18
ZSM-5-deAl-1 / 100	240	1.5	99	99	S18
Hierarchical ZSM-5 (M1)	300	1.1	86	80	S19
Hierarchical ZSM-5 (M2)	300	1.1	99	86	S19
SiAlPO-34	230	0.3	96	91	S5
MgSiAlPO-34	230	0.3	99	94	This work

a) Conversion of ethanol, b) Selectivity to ethylene.

Kinetic analysis

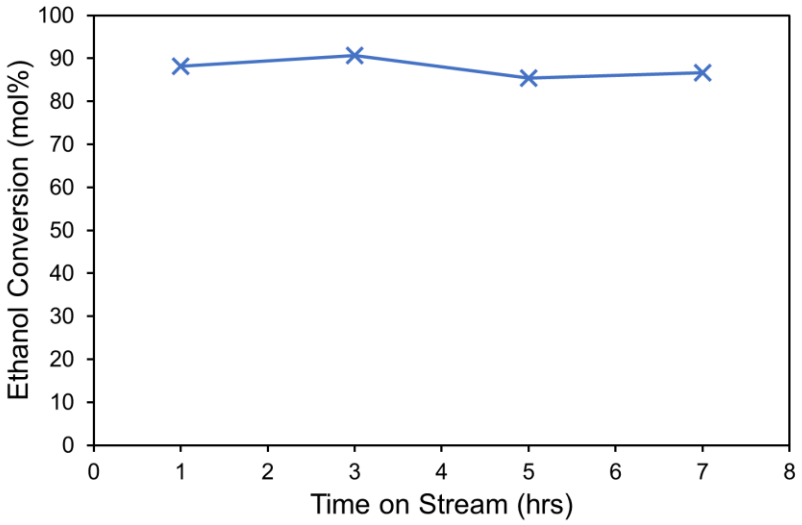

Figure S5: Showing the stability of MgSiAlPO-34 after 7 hours on stream at 185 ^oC, 0.3 g catalyst, He carrier gas = 25 mL/min, WHSV = 0.3 hr^{-1}. Initial conversion after 1 hour is 88.2 mol%, after 7 hours this is still at 86.7 mol%, confirming its stability.

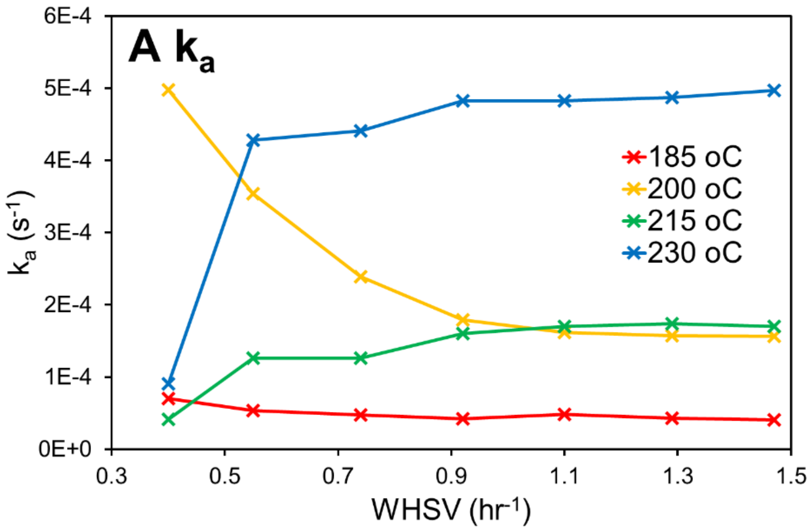

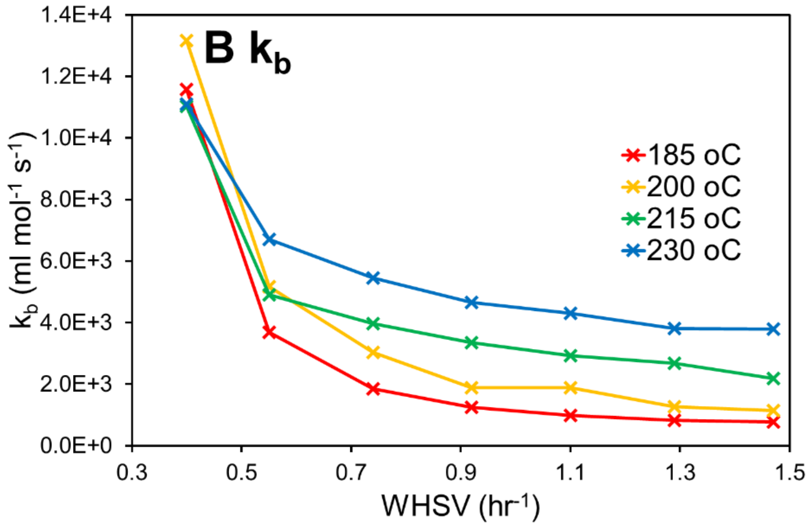

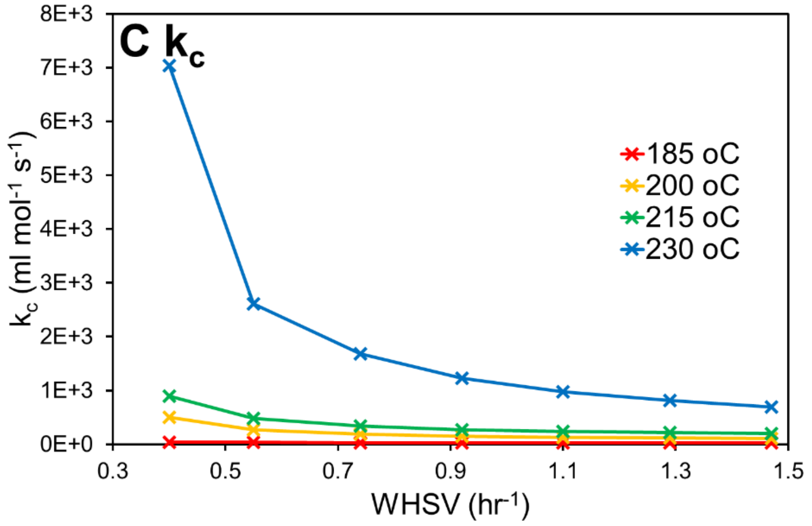

Figure S6: Variations in rate constants of the different reaction pathways for varying WHSVs over different temperatures for A) k_a, b) k_b and C) k_c.

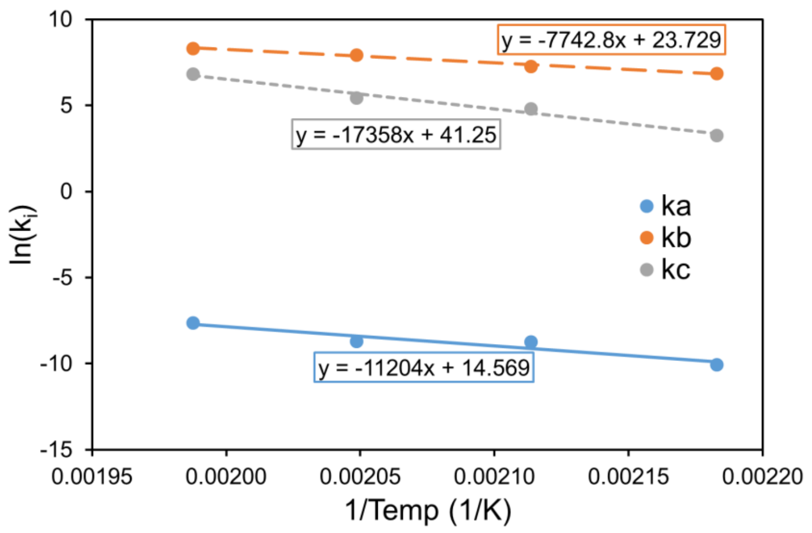

Figure S7: Arrhenius plot of the calculated average rate constants across the 0.92-1.47 hr^{-1} WHSVs cases to derive the activation energy.

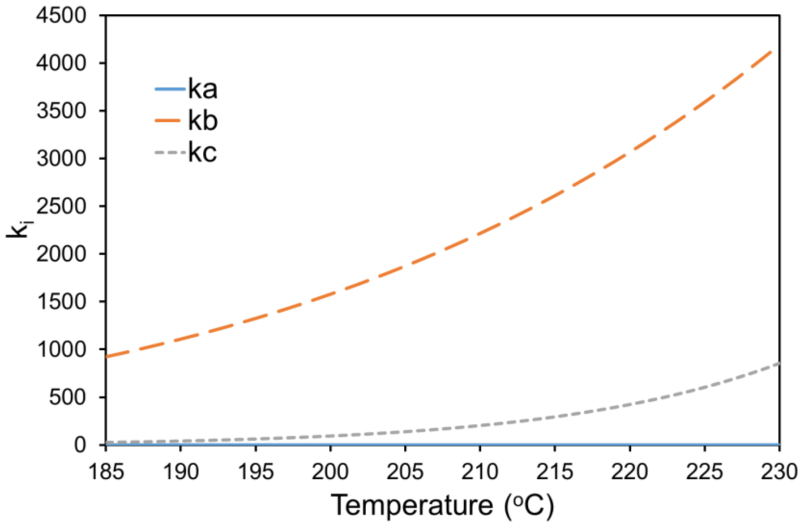

Figure S8: Variation in rate constant values for the three reaction steps as a function of temperature in the kinetically limiting regime; WHSV > 0.92 hr^{-1}.

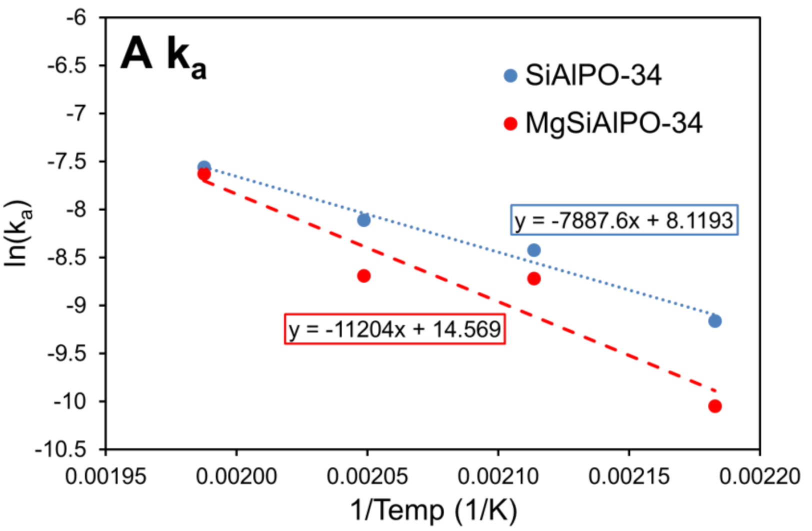

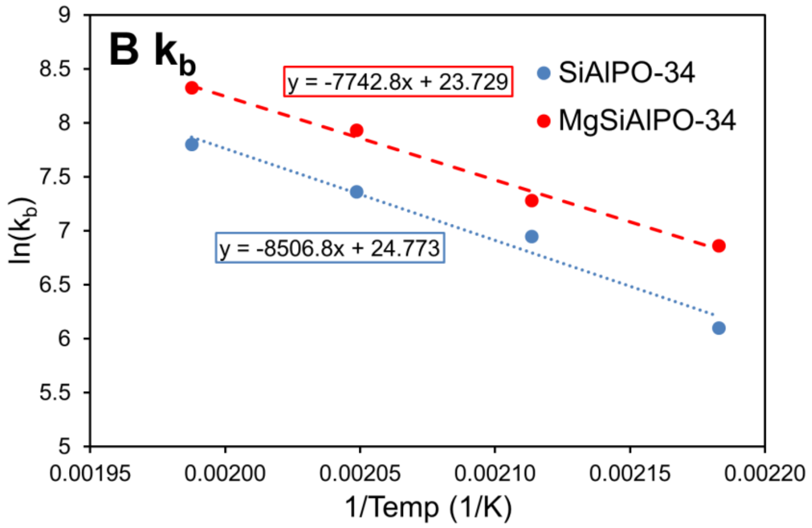

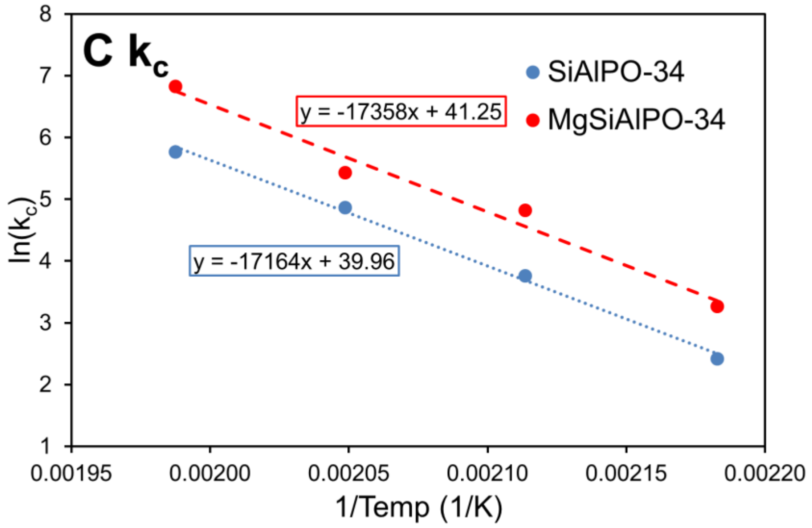

Figure S9: Arrhenius plot comparing the calculated average rate constants for both the MgSiAlPO-34 and the SiAlPO-34 catalysts in the kinetically limited regime; WHSV > 0.92 hr^{-1} for A) k_a, B) k_b and C) k_c.

Computational fluid dynamics data

Table S8: Exiting molar concentrations and mole fractions for the MgSiAlPO-34 for the 1.47 WHSV hr^{-1} case, at varying temperatures

		Molar concentration (mol/ml)				Mole fractions			
		C_2H_5OH	C_2H_4	$C_4H_{10}O$	H_2O	C_2H_5OH	C_2H_4	$C_4H_{10}O$	H_2O
**185 °C**	**Computational**	3.08E-07	5.31E-07	2.27E-06	2.80E-06	5.22	8.98	38.41	47.39
	**Experimental**	4.41E-07	3.50E-07	2.80E-06	3.15E-06	6.55	5.19	41.53	46.72
**200 °C**	**Computational**	3.23E-07	1.36E-06	1.78E-06	3.14E-06	4.89	20.64	26.92	47.55
	**Experimental**	4.97E-07	1.33E-06	2.28E-06	3.61E-06	6.44	17.26	29.52	46.78
**215 °C**	**Computational**	2.86E-07	2.50E-06	1.14E-06	3.64E-06	4.40	30.29	17.51	47.80
	**Experimental**	4.20E-07	2.04E-06	1.96E-06	4.00E-06	4.99	24.18	23.33	47.51
**230 °C**	**Computational**	2.09E-07	3.47E-06	6.11E-07	4.08E-06	2.50	41.44	7.31	48.75
	**Experimental**	3.09E-07	3.97E-06	1.05E-06	5.02E-06	2.99	38.37	10.14	48.51

References

[S1] B. M. Fung, A. K. Khitrin and K. Ermolaev, *J. Magn. Reson.*, 2000, **142**, 97.

[S2] S. Hopps, S. Sahle, R. Gauges, C. Lee, J. Pahle, N. Simus, M. Singhal, L. Xu, P. Mendes and U. Kummer, *Bioinformatics* **2006**, *22*, 3067-3074.

[S3] M. Zhang and Y. Yu, *Ind. Eng. Chem. Res.*, 2013, **52**, 9505.

[S4] V. V. Bokade and G. D. Yadav, *Applied Clay Sci.*, 2011, **53**, 263.

[S5] M. E. Potter, L. M. Armstrong and R. Raja, *Catal. Sci. Technol.* **2018**, *8*, 6163-6172.

[S6] S. Ergun, *Chem. Eng. Prog.*, 1952, **48**, 89.

[S7] N. K. Kochar, R. Merims and A. S. Padia, *Chem. Eng. Prog.*, **1981**, *77*, 66–70.

[S8] Tardy, M., Solvay Indupa will produce bioethanolbased vinyl in Brasil & considers state of the art power generation in Argentina. Polyvinylchloride (PVC) derived from sugar cane and salt, http://www.solvay.com/EN/NewsPress/Documents/2007/20071214_ColomboII_EN.pdf. Cited December 14, 2007.

[S9] Z. Gao, M. Y. He and Y. Y. Dai, *Zeolite Catalysis and Separation Technology, Beijing: China Petrochemical Press*, 1999.

[S10] *Focus Catal.*, **2012**, *9*, 3–4.

[S11] L. R. Pan and H. X. Li, *Petrochem. Technol.*, **1985**, *14*, 154–157.

[S12] M. E. Potter, M. E. Cholerton, J. Kezina, R. Bounds, M. Carravetta, M. Manzoli, E. Gianotti, M. Lefenfeld and R. Raja, *ACS Catal.*, **2014**, *4*, 4161-4169.

[S13] D. Varisli, T. Dogu and G. Dogu, *Chem. Eng. Sci.*, **2007**, *62*, 5349-5352.

[S14] K. Ramesh, L. M. Hui, Y. F. Han and A. Borgna, *Catal. Commun.*, **2009**, *10*, 567-571.

[S15] T. K. Phung and G. Busca, *Catal. Commun.*, **2015**, *68*, 110-115.

[S16] T. K. Phung, L. P. Hernámdez, A. Lagazzo and G. Busca, *Appl. Catal. A: Gen.*, **2015**, *493*, 77-89.

[S17] G. Chen, S. Li, F. Jiao and Q. Yuan, *Catal. Today*, **2007**, *125*, 111−119.

[S18] C. Y. Wu and H. S. Wu, *ACS Omega*, **2017**, *2*, 4287-4296.

[S19] K. A. Tarach, J. Tekla, W. Makowski, U. Filek, K. Mlekodaj, V. Girman, M. Choi and K. Góra-Marek, *Catal. Sci. Technol.*, **2016**, *6*, 3568-3584.
